# Linking Activity Theory Within User-Centered Design: Novel Framework to Inform Design and Evaluation of Adverse Drug Reaction Reporting Systems in Pharmacy

**DOI:** 10.2196/43529

**Published:** 2023-02-24

**Authors:** Joel Fossouo Tagne, Reginald Amin Yakob, Rachael Mcdonald, Nilmini Wickramasinghe

**Affiliations:** 1 School of Health Sciences and Biostatistics Swinburne University of Technology Melbourne Australia; 2 Centre for Health Analytics Murdoch Children's Research Institute Melbourne Australia; 3 MedTechVic Swinburne University of Technology Melbourne Australia; 4 Pharmaceutical Society of Australia Sydney Australia; 5 Department of Nursing and Allied Health, Occupational Therapy Swinburne University of Technology Melbourne Australia; 6 Iverson Health Innovation Research Institute Swinburne University of Technology Melbourne Australia; 7 Epworth Healthcare Melbourne Australia

**Keywords:** pharmacovigilance, adverse drug reaction, pharmacist, user-centered design, activity theory

## Abstract

**Background:**

Adverse drug reactions (ADRs) may cause serious injuries including death. Timely reporting of ADRs may play a significant role in patient safety; however, underreporting exists. Enhancing the electronic communication of ADR information to regulators and between health care providers has the potential to reduce recurrent ADRs and improve patient safety.

**Objective:**

The main objectives were to explore the low rate of ADR reporting by community pharmacists (CPs) in Australia, evaluate the usability of an existing reporting system, and how this knowledge may influence the design of subsequent electronic ADR reporting systems.

**Methods:**

The study was carried out in 2 stages. Stage 1 involved qualitative semistructured interviews to identify CPs’ perceived barriers and facilitators to ADR reporting. Data were analyzed by thematic analysis, and identified themes were subsequently aligned to the task-technology fit (TTF) framework. The second stage involved a usability evaluation of a commercial web-based ADR reporting system. A structured interview protocol that combined virtual observation, think-aloud moderating techniques, retrospective questioning of the overall user experience, and a System Usability Scale (SUS). The field notes from the interviews were subjected to thematic analysis.

**Results:**

In total, 12 CPs were interviewed in stage 1, and 7 CPs participated in stage 2. The interview findings show that CPs are willing to report ADRs but face barriers from environmental, organizational, and IT infrastructures. Increasing ADR awareness, improving workplace practices, and implementing user-focused electronic reporting systems were seen as facilitators of ADR reporting. User testing of an existing system resulted in above average usability (SUS 68.57); however, functional and user interpretation issues were identified. Design elements such as a drop-down menu, free-text entry, checkbox, and prefilled data fields were perceived to be extremely useful for navigating the system and facilitating ADR reporting.

**Conclusions:**

Existing reporting systems are not suited to report ADRs, or adapted to workflow, and are rarely used by CPs. Our study uncovered important contextual information for the design of future ADR reporting interventions. Based on our study, a multifaceted, theory-guided, user-centered, and best practice approach to design, implementation, and evaluation may be critical for the successful adoption of ADR reporting electronic interventions and patient safety. Future studies are needed to evaluate the effectiveness of theory-driven frameworks used in the design and implementation of ADR reporting systems.

## Introduction

### Background

The use of medications may result in adverse drug reactions (ADRs) that may increase the risk of patient morbidity and mortality. The timely reporting of ADRs to regulators may contribute to patient safety by facilitating information gathering on drug safety data [1]. Worldwide ADR-related hospital admission ranges from 3.6% to 15.6% [2,3]. In Australia, ADR-related hospital admissions are estimated at 7.2% to 11% where 50% of ADR-related hospital admissions are preventable [4-6]. Furthermore, in Australia, medication-related problems account for approximately AUD $1.4 billion (US $937,440,000) per annum, that is, 15% of the total Australian Pharmaceutical Benefits Scheme [7]. The reporting of ADRs to regulators is important for postmarketing surveillance, quality improvements, and drug safety research, but they are vastly underreported [8]. The challenges to ADR reporting include lack of awareness by health professionals (HPs) or consumers, lack of time or financial incentives by HPs, legal implications, attitudes of the reporters (eg, reduced motivation, and lack of efficient/user-friendly reporting systems for clinicians) [9-11]. Poor documentation and reporting between care providers or across health care settings are a major roadblock to patient safety from known ADRs [12]. Critical information regarding ADRs or serious health conditions (eg, COVID-19) may remain elusive to HPs that prescribe and dispense drugs or regulators who govern or approve new drugs [12]. Furthermore, we previously identified that there is also substantial interinstitutional variability in the standards of ADR reporting among individual primary health care facilities by conducting a scoping review [13]. Therefore, re-exposure to harmful drugs can be potentially avoided by improving health care systems and medication supply practices [14].

Digital documentation and reporting of ADRs currently occur within web-based reporting systems hosted by regulatory organizations (eg, the Therapeutics Good Administration or Surveillance of Adverse Events Following Vaccination In the Community) [15,16].

Although these existing reporting systems are structured and standardized, they are perceived as cumbersome and time-consuming to navigate [1,16]. Therefore, ADR reporting within these websites is disconnected from the needs of HPs, and immediate patient care-related activities may supersede the data request of external agencies [12,17]. If ADR reporting was refocused to meet the patient safety information needs of the HPs who recognize, treat, and encounter new ADRs at the point of care, clinicians may be more willing to document and report these harmful events [12,18]. Digital health interventions such as e-prescribing, e-medical records, digital health records, and health mobile apps have been introduced in the last decade [19,20]. These interventions can promote efficiency across health processes, enhance patient satisfaction, and save costs [8]. Therefore, the uptake of electronic medical records provides opportunities for ADR reporting to be integrated into point-of-care systems [17]. Despite the promise that such technologies hold for integrated patient care and safety, their uptake among HPs has been slow, and this is likely due to assumptions that govern their design [12].

A 2020 systematic review of interventions to improve ADR reporting concluded that there was scope to include community pharmacists (CPs) to improve ADR reporting [9]. These findings were also consistent in other reviews [16,19]. The implementation of digital systems to support reporting by care providers and designing systems within the clinician workflow have been highly regarded [16,19,21]. To date, knowledge gaps exist regarding the practice and reporting of ADRs reporting by CPs in Australia [18]. To our knowledge, only 1 previous study explored the knowledge and perspective of CPs toward ADR reporting in Australia and found that 43% (n=101) of respondents agreed that a lack of time within their professional practice limited their reporting of ADRs and 65% (n=150) agreed that remuneration would encourage them to report ADRs [18]. Integration of autopopulation features within the dispensing software was identified as an efficient way to facilitate ADR reporting by CPs [18].

### ADR Surveillance Systems

The safety surveillance of medications may be passive or active [22]. Active surveillance systems systematically monitor particular patient encounters to seek information about ADRs (eg, artificial intelligence), whereas passive surveillance systems provide opportunity for point-of-care providers to confidentially and voluntarily report ADRs [22]. In Australia, the GuildCare (GuildLink) is a passive surveillance system available in Australian community pharmacies [18,23]. The system was released in June 2014, allowing CPs to record and report ADRs directly to the Therapeutic Goods Administration (TGA) [8,18,24]. Soon after it was released (ie, June to September 2014), there was an increase in the rate of ADR reporting via the Guildlink portal [8]. The TGA received ADR reports nearly as high as that for the entire year of 2013, suggesting the system may have been well received by CPs [18]. However, despite the positive start, the numbers declined again in 2015, indicating there may be a need for constant reminders to maintain ADR reporting rates, and continuing system evaluation requirements [18]. A systematic review of adverse event reporting information systems found wide variation in the variety and type of data collected [25]. In addition, these reporting systems did not report pilot testing to ensure there was succinct, user-friendly, relevant, and correct interpretation of electronic fields by care providers prior to their implementation [25,26]. Implementing new systems without pilot testing and refining may fall short of their expected goals due to systems’ architecture constraints or design failures that could have been identified and resolved prior to their final build [27]. User-centered design (UCD) is a framework that places users (eg, CPs) at the center of the design process from the initial stages of planning, designing the system requirements, evaluating, and deployment of the final product [28]. It involves the influence of end users during the design processes and has been shown to contribute to the acceptance, adoption, and success of systems [29]. The core principles of UCD include: (1) understanding and specifying the context of use, (2) specifying the user including the organizational requirements, (3) producing design solutions, and (4) evaluating designs against requirements (Multimedia Appendix 1) [28,29]. Poor uptake of adverse event reporting systems by HP can occur when the system is designed without or with limited clinician input while prioritizing organizational data needs [12,30]. To optimize both the effectiveness and usefulness of these interventions, usability and acceptance are essential.

To date, there have been no reporting on factors affecting the implementation and adoption of pharmacovigilance (PV) systems in Australian community pharmacies and other primary care settings [16]. A key challenge to the successful utilization of any new system lies in strategies that drive uptake and adoption [28]. A recent 2022 systematic review concluded that future interventions should include a comprehensive multifaceted approach to improve the quantity and quality of ADR reporting [19]. A comprehensive multifaceted approach includes incorporating digital technologies with additional strategies that specifically address the key factors of a behavioral change framework [19]. The use of a behavioral change framework to investigate ADR reporting has previously been described [31,32]. As newer innovations emerge and digital technologies continue to transform health care management, several barriers still remain [33]. A recent Australian study reported the use of a theoretical domains framework together with a technological intervention as a strategy to facilitate ADR reporting by clinicians in hospitals [19,31]. However, the perspective of pharmacists working in community pharmacies is lacking [18,21]. The benefits of such digital systems are presumed to follow logic, and assumptions are that end users and clinical settings will adapt to the new technologies [12]. After identifying such assumptions and the potential detrimental impact on patient safety, our objectives were to understand why ADR reporting is low among CPs and examine barriers to reporting within their existing systems. This paper then provides insight into the application of activity theory (AT) from the fields of human behavior and information‐communication as a framework to inform the evaluation or design of user-centered ADR reporting systems.

## Methods

### Overview

This was an exploratory study, with the underlying epistemology stemming from a social-constructivist paradigm, as the goal was to understand the knowledge constructed through CP’s practice lens. The study was predominantly qualitative, with some quantitative data that served as descriptive statistics. The study was carried out in 2 stages. Stage 1 was to gain a deeper understanding of the problem of low ADR reporting among CPs through understanding the “users” (CPs) and their social or environmental milieu. The individual results of stage 1 have previously been published [34]. Given the decline in electronic reporting through the Guildlink portal as discussed above, stage 2 was to evaluate the usability of a commercially available ADR reporting system (GuildCare system) to understand what attributes and features facilitate or prevent reporting (submitted for publication). Purposive sampling was used to select eligible participants working in community pharmacies listed on the Pharmacy Guild of Australia and Health direct website. Participants agreed to participate in a 25- to 60-minute recorded virtual interview. As we have submitted stage 2 for publication and previously published stage 1 of this work [34], in this paper, we have focused on the research results used to develop and propose our ADR reporting design and evaluation framework.

### Ethics Approval

Before conducting the interviews, all participants provided informed written consent to participate in the study and were advised that the provided information may be deidentified and used for publication. Participants’ demographic data were collected by using a self-administered questionnaire, which was attached to the consent form. All procedures followed were in accordance with Australia’s National Statement on Ethical Conduct in Human research (2018). The study was approved by the Swinburne University of Technology Human Research Ethics Committee (Ref: 20214304-6249).

### Stage 1: Understanding the Problem (Low ADR Reporting), User (CPs), and the Context (Community Pharmacy)

A qualitative study with individual interviews was conducted with CPs working across Victoria, Australia, between April 2022 and May 2022. A semistructured interview guide was used to identify CPs’ perceived barriers and facilitators to ADR reporting. Because this research also sought to explore strategies to implement innovative technologies to facilitate ADR reporting, the task-technology fit (TTF) model offered guidance when developing the semistructured interview questions and categorizing identified themes [8].

Task characteristics refer to the attributes of a task that can be executed using information communication technologies for the purpose of satisfying work practice needs (eg, dispensing a prescription). Tasks can vary in a number of dimensions including task nonroutineness, task interdependence, and time criticality. The users’ workflow and environment are also key considerations when assessing the “Fit” [35].

Technology characteristics refer to the technology tool used by individuals in carrying out their tasks. The aspects of technology tools may influence technology utilization and users’ perceptions. The TTF considers the importance of fitting the functionality and attributes of technology used, to the demands imposed by individual needs. Technology tools can either be hardware or software [36].

Data were analyzed by thematic analysis. Themes were constructed from the CPs’ reported barriers and facilitators. Thematic analysis began once interviews were complete using NVivo 12 software (QSR International). Initially, open codes were generated inductively from the participants’ descriptions of their experiences in reporting ADRs and the barriers or facilitators to reporting. The final analysis for this study focused on the key themes constructed from the interviews and was subsequently mapped into the TTF model. Data concordance was verified by coauthors NW and RM, who are both experienced in public and digital health research. Key themes were discussed with the research team that included clinicians with expertise in the quality use of medicine and drug safety. Interviews concluded when no additional themes could be identified and mapped to the TTF theoretical framework

### Stage 2: Usability Evaluation of an Online Reporting System (GuildCare)

**Figure 1:**
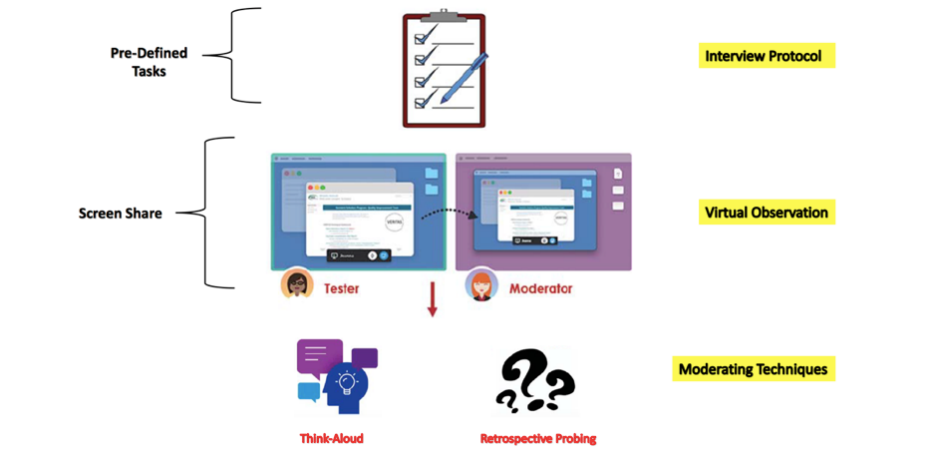
Summary of the system usability testing approach.

A structured interview protocol (Figure 1) was developed that was designed to evaluate both usefulness and satisfaction; the interview protocol leveraged think-aloud moderating techniques (assessing usefulness), retrospective questioning about user satisfaction, and administration of the System Usability Scale (SUS; assessing satisfaction).

The SUS is a flexible questionnaire designed to assess any technology and is relatively quick and easy to complete [14,37]. It consists of 10 statements that are scored on a 5-point scale, 1=strongly disagree to 5=strongly agree, with the final scores (after transformation of the scores) ranging from 0 to 100 [38]. A higher score may indicate better usability. As a general rule, a system that has a score above 68 has acceptable usability; a lower score means that the system needs more scrutiny and continued improvement [14].

Usability testing relied on participants’ verbal communication and virtual observation through screen sharing [39]. During the interview, participants were directed to complete an ADR report scenario using a semistructured interview protocol. Thematic analysis began once interviews were completed using NVivo 12 software and was performed by 2 members of the team. The key themes were discussed among the research team that included a pharmacist and an engineer with experience in digital health. Interviews concluded when no additional themes relating to the research question could be found.

## Results

### Stage 1

In total, 12 CPs were interviewed. The themes identified spanned both task and technology aspects of the TTF sociotechnical framework. From the data, we identified the theme “lack of time” as a barrier to ADR reporting, which is consistent with previous studies in community pharmacy [18,40]; however, by using the TTF model, we were able to further analyze this theme by contextualizing what different CPs generally mean when they say, “lack of time to report.”

When CPs reported “lack of time,” this was either the requirement to stop performing regular duties, for example, clinical tasks and attend to the ADR reporting process or they were referring to the prolonged duration when “completing a reporting form,” for example, a digital regulatory reporting form. Within the first context, “lack of time” may be considered as a dependent variable, influenced by environmental factors, for example, the work environment or lack of support staff. In the second context, the CPs referred to the cumbersome web-based reporting forms. The identified barriers and suggested intervention strategies to ADR reporting is divided into 2 broad categories, corresponding to components of the TTF and is listed in Textbox 1.

**Community pharmacists’ reported barriers and facilitators to adverse drug reaction reporting aligned to the task-technology fit.**

- Barriers corresponding to the task-technology fit framework
  Task: Lack of knowledge to adverse drug reaction reporting, time constraints, lack of financial incentives, lack of organizational support for adverse drug reaction reporting, and preference to refer consumers to physicians.
  Technology: Low awareness to adverse drug reaction reporting systems, fragmented reporting systems, and inadequate organizational IT infrastructure.
- Facilitators corresponding to the task-technology fit framework
  Task: Enhancing community pharmacists knowledge and awareness of adverse drug reactions, environmental restructuring and financial incentives for adverse drug reaction reporting, education, and empowering consumer reporting.
  Technology: Workflow-integrated adverse drug reaction reporting technology systems, feedback provision to community pharmacists on the reported adverse drug reactions, and promoting consumer adverse drug reaction reporting.

### Stage 2

**Figure 2:**
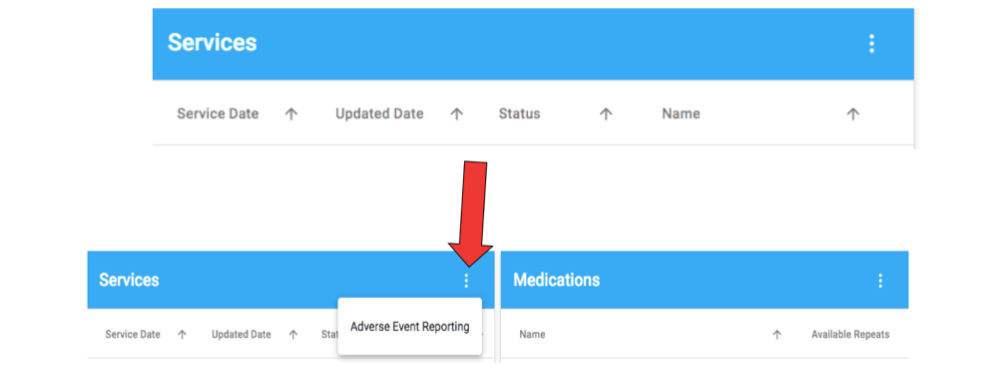
Main screen to select and start an adverse event report.

**Figure 3:**
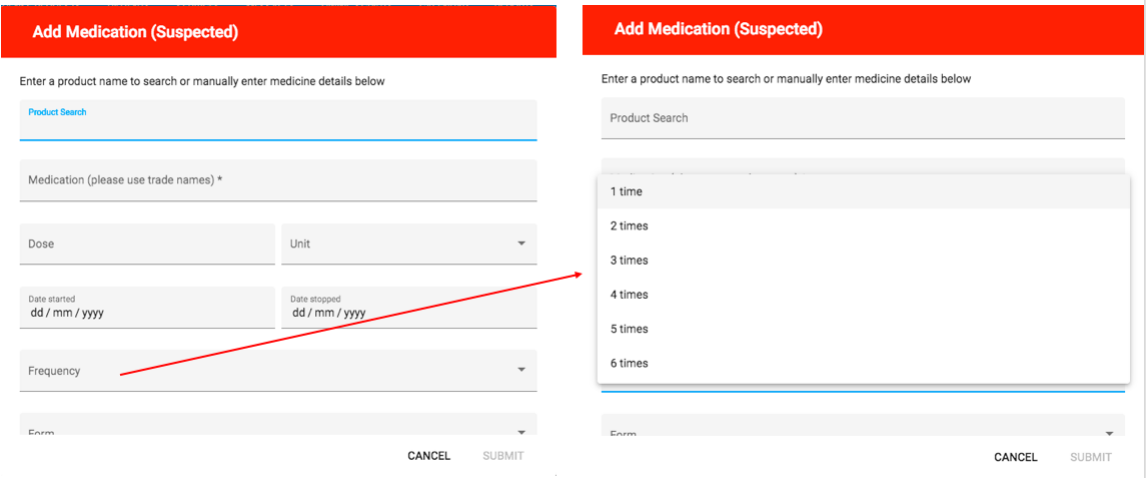
Main screen to add suspected medication displaying frequency times.

In total, 7 CPs participated in the usability study. The system was perceived to have above average usability (SUS 68.57). Despite this, the use of a structured approach to usability testing identified themes that would have been overlooked by the results of the SUS alone. For example, when observing CPs navigate the system, all participants struggled to begin the task (ie, the ADR report) when they initially logged-in to the system. Despite the presence of 3 dots on the main user screen to begin the report, participants felt it was not clearly visible and lacked clarity (Figure 2). When adding the suspected medication participants were unsure about the frequency field (Figure 3), that is, 1, 2, or 3 times ongoing:

not sure whether these options refers to the initial medication dose regimen or maybe the number of doses that had been taken or even the number of times the ADR was experienced.

CP4

In addition to this, participants also struggled to complete and submit the ADR report and were confused as to why the form could not be submitted. In 5 of 7 interviewees, participants were verbally guided by the moderator to review and search for potential compulsory fields with missing data input that could prevent the form from being successfully submitted. This was not self-evident to the participants, adding more time to complete the report. Design elements such as a checkbox, drop-down menu, free-text entry, and prefilled/autopopulated data fields were perceived to be extremely useful for navigating the system and facilitating ADR reporting. Identified and reported themes have been divided into barriers and facilitators (Textbox 2).

**Observed and participant-reported barriers and facilitators.**

**Barriers**

- Navigations (accessing and submitting the report; workflow, eg, required multiple steps)
- Irrelevant data fields
- Minimum required data
- Lack of system integration (web-based vs within dispensing system)
- Lack of interoperability (sharing with allied health)
- Length (number of data fields/questions)

**Facilitators**

- Drop-down menu
- Auto-filled sections
- Search options (eg, medications)
- Combination of checkboxes, drop-down menu, and free-text entry
- Direct submission of report to the Therapeutic Goods Administration
- Succinct list and relevant to setting

## Discussion

### Overview

The study objective was to understand why ADR reporting is low among CPs and examine the barriers or facilitators to ADR reporting within the existing system. The knowledge of barriers and facilitators to ADR reporting may inform the design of electronic ADR reporting systems that are fully integrated within the CPs workflow. Ideally, such systems will be used by clinicians (CPs) to facilitate ADR documentation at the point of care. Furthermore, they will allow for information sharing with regulators, among care providers and across health sectors to prevent unintentional re-exposures of patients to harmful drugs. This study sought to address a methodological gap in the way that ADR reporting systems have previously been conceptualized, designed, and implemented. Poor usability can arise from existing adverse event reporting systems that have been designed at a distance from users, with limited clinician input, prioritizing organizational data needs [12,30]. Previous research in Australia have reported that CPs are simply not using these systems, as the act of reporting is perceived as secondary to clinical care delivery, and systems are time-consuming, cumbersome to use, and are not integrated into current electronic information systems [18,19]. These findings have also been reported across multiple international jurisdictions [41]. The vast majority of ADRs remain underreported and are not reflected in the current health data that are used by regulators, including research organizations that examine drug safety [19,21,42]. Preventable ADRs may go unaddressed to the detriment and cost of health consumers, health care systems, and taxpayers [12].

### Theory-Driven Intervention Design

Prior research on ADR underreporting have suggested initiatives to improve reporting predominantly focusing on the users and have rarely scrutinized the systems in place [16,19]. The results of these studies suggest shortcomings that include poor user knowledge, lack of awareness, clinical priorities, incentives, and workplace culture [17]. Prior studies to improve ADR reporting have not questioned the data-centric orientation of electronic reporting systems and have not explored systems shortcomings, or proposed ways to redesign reporting systems to facilitate reporting, complement clinical care while meeting the data needs of regulators [9,19]. Globally, studies have discussed interventions to improve ADR reporting among health professionals; however, the suggested interventions are of a general nature [9,16], without an evidence-based theoretical framework or adequate assessment of the end user needs [31,32]. An intervention applied in 1 setting may not be appropriate for another health setting, and there is therefore a need for an evidence-based method to guide the selection and implementation of relevant interventions [29,32].

The behavior of HPs in reporting ADRs can be influenced by different factors, including individual characteristics and those that involve the external environment [43]. Therefore, it is necessary to understand ADR reporting behavior of CPs using a well-defined theoretical approach [9,24]. In our study, we found the application of the TTF (stage 1) to be useful in our data analysis and understanding of the problem [34]. Where previous studies have reported “lack of time” as a barrier to ADR reporting [18], we were able to apply context, to understand what different CPs meant by the common phrase “Lack of time to report.” Nonetheless, the first context “lack of time” was a dependent variable, influenced by environmental constraints, for example, a lack of support staff, while in the second context, the CPs were referring to the cumbersome and time-consuming process of digital reporting web forms. The UCD approach begins with gaining a clear and thorough understanding of the users and task analysis, including the context of use, which is key to the implementation and adoption of the system [29]. Failure to understand the fundamental needs of end users when developing ADR reporting interventions may lead to reduced system usage and negatively impact patient safety. The TTF model has been applied in health care settings where businesses require technology solutions [44].

### Limitation of TTF in ADR Interventions Design

The use of the TTF theoretical model to support our research inquiry may have limited the exploration of other important factors. For example, in stages 1 and 2, some CPs made comments such as “I think doctors are responsible for ADR reporting” or “we can report, but I’m sure anyone can report, including customers.” While in our study we determined the uncertainty around the responsibilities for ADR reporting as a lack of knowledge associated with the “task” (TTF), it may also suggest a lack of task ownership or task responsibility by the CPs, which could impact intervention design and successful implementation. In his theory on systems of professions, Abbott argued that individuals of professions generally define their jurisdictions, that is, the link between a profession and its work, by claiming exclusive rights over particular tasks [45]. However, in Australia, ADR reporting is a task that is not exclusive to CPs per se; instead, this task is conferred upon them and other HPs by the regulators (eg, TGA). CPs are not exclusively responsible for undertaking ADR reporting; it is a shared task between CPs, doctors, allied health professionals, health consumers, and the public [8]. Furthermore, it is a voluntary act to be performed for the purposes of advancing drug safety knowledge and patient safety. Therefore, when considering interventions or designing new systems, it would be beneficial to explore what happens to tasks like ADR reporting that are shared and not specifically claimed by a professional group. The ideal situation for drug safety monitoring would be that all HPs claim responsibility for the task and report. However, there is a blur in the boundaries of task allocation. This may result in the potential for ADR reporting to be ignored across HPs including CPs and could be a reason why the ADR reporting rate declined again after the initially reported increase in 2014 when the GuildCare ADR system was released [18].

### AT as a Conceptual Lens of Analysis in ADR Reporting

Given the limitations found in the TTF, we undertook a further review of the various sociotechnical theories that could encompass the multifaceted and dynamic contexts involved in human decision and ADR reporting. The alternative theories explored included theories of planned behavior, technology acceptance model, and unified theory of acceptance and use of technology model. While these theoretical frameworks explore human behavior and use intention [46,47], they do not assess human interaction within the entire work system (eg, teams and organizations) as discussed in the limitations of using the TTF framework (ie, task ownership associated with ADR reporting). However, following further exploration, the constructs of AT were found to be fitting. AT is a descriptive approach that explains human practices in the social context (Multimedia Appendix 2) [8]. This theory considers the viewpoints and behaviors of users in a social context, originally based on the work of Vygotski and the study of cultural-historical psychology [48,49]. The AT framework uses “activity” as the fundamental unit to study human interaction [29]. The activity (what people do) is reflected through actions as people interact with their environment, thus providing a richer analysis of the user’s needs, context, and the direct or indirect environment [29]. The components of activity include subject, object, tools, rules, community, division of labor, and outcomes [49].

Reflecting on the activity model (Multimedia Appendix 2), researchers, designers, and developers of electronic ADR reporting systems may define the different constructs as follows: AT can be used to understand the interaction among the subjects (HPs or consumers/patients) and the objects (activities and processes involved in documenting and reporting ADRs). The tools in this study are the reporting systems (eg, GuildCare reporting systems) used to record and report ADRs to the regulators or share information with members of the community. The rules guiding these activities include the organizational, jurisdictional, or federal laws regarding ADR reporting [50]. The community that takes part in these activities may include pharmacists and other health professionals, patients, and regulators. Within these activities, work (PV) is divided among the community [29,48].

### Linking AT Within UCD

Human-computer interaction is a complex interdisciplinary field, concerned with design, implementation, and evaluation [29]. As such, we hope to make a novel attempt to operationalize AT as a theoretical lens for a UCD framework to support improvements or the development of new electronic PV interventions in pharmacy. UCD (Multimedia Appendix 1) begins with a thorough understanding of the needs and requirements of the users (CPs). Analyzing the interaction among potential users is also very important, and based on the UCD approach, establishing the context in which users may use the system should be defined at the beginning.

Using AT, the user needs and requirements can be investigated to provide a structured and richer understanding of the subjects’ (users) needs as well as their related activities (eg, ADR reporting or clinical tasks). These activities can then be separated into subjects, tools (intervention), and objects (outcome). A usable system not only understands the needs of the user but also understands a user’s situation (ie, the context and environment) [51]. Therefore, AT can help to examine the user’s environment, including their social or cultural milieu. The organization requirements can also be explored using the constructs from AT with the UCD framework, which may also be useful in evaluating acceptance [29]. Furthermore, the design addresses the whole user experience, not solely focusing on the usability of the system but also ensuring a positive user experience [51].

The user experience may be evaluated through the use of questionnaires and interviews that probes end user experiences after using a system [52]. In stage 2 (usability testing), collectively, the CPs perceived the system to have above average usability (SUS 68.57). However, through our structured approach, combining virtual observation, think-aloud, and retrospective probing with the SUS, we observed functional and user interpretation issues impacting user experience that would have been easily overlooked if we had simply relied on the SUS results or interviews (Textbox 2); for example, difficulties in accessing and submitting the reporting forms or confusions over the intent of data fields. Considering HPs already face time constraints, factors impacting their time may be perceived as an additional documentation burden, causing reduced adoption and affecting patient safety. Therefore, it is important to note that users may have different perceptions, understanding, and expectations of a system, which may affect how they interact with the system and may not always be reflected in surveys or interviews [51]. Furthermore, human activity is directly influenced by social, cultural, and historical context, which adds further complexity [53]. Applying AT to UCD may help provide more emphasis on the user’s interaction and requirements. This may also help to bridge any gaps by adapting contextual information to the user’s situation and needs [53]. Based on our findings, we propose a framework for leveraging AT within UCD in ADR reporting (Multimedia Appendix 3). The establishment of this framework may support the requirement stage (user or organizations) of UCD. It may allow stakeholders to gain a comprehensive understanding of the context and the user needs prior to system design (Multimedia Appendix 3, steps 1 and 2). Furthermore, the proposed framework may also be used during system evaluation and iterations.

### Future Strategies to Improve ADR Reporting

There is great potential to leverage recent developments in digital technologies to improve ADR reporting [8,19]. Digital technologies are widely available in the areas of automation, data mining, and signal detection of ADRs [8]. For example, in Australia, an active vaccine safety surveillance system integrated with national surveillance networks was successfully linked with a cloud-based pharmacy vaccination recording system to develop an automated active vaccine safety surveillance system for community pharmacies [54]. This was introduced in response to the COVID-19 pandemic and automatically reports immunizations directly to the Australian Immunisation Register [54]. Furthermore, increased advancements have been made in the area of artificial intelligence and machine learning in the detection of ADRs, with 1 study showing an 80% success rate in automated ADR detection in the hospital setting [55]. However, these ADR surveillance systems are different from passive surveillance. These involve manually reporting ADRs and are dependent on behavioral changes from the clinician, organizational or workplace structures, and operational/IT infrastructures [22]. Previous interventions such as education, reminders, feedback, and so forth, have only been temporarily effective in improving ADR reporting rates with the effect diminishing substantially within 6-12 months after implementation [18,56-58]. Furthermore, these interventions may need continuous maintenance to improve ADR reporting rates, which may be time-consuming and expensive [19]. Studies investigating ADR underreporting have primarily focused on knowledge and attitudes, advocating for interventions targeting provider behaviors [17].

However, in practice, the successful implementation and adoption of a new technology often hinge on how well these systems are integrated into organizational and clinical practice, and whether they meet the needs and expectations of the end users [12,29]. Applying theory-driven and best practice approaches (eg, our proposed AT and UCD framework) to systems design, implementation, and evaluation may bring more rigor, robustness, and accountability to new ADR surveillance interventions.

### Limitations

The findings reflect the activities and opinions of CPs working within the settings where we were able to conduct the study. CPs’ responses may have been shaped by the organizational context for reporting ADRs within the jurisdiction. We used purposive sampling that could have resulted in selection bias. The sample size may be seen as a limitation; however, there were varied opinions from many who do not regularly report ADRs, suggesting the strength of socially desirable bias may not be too strong. The study focused its inquiry using the TTF theoretical model, which may have limited exploration of other important factors, as discussed earlier. We spoke with CPs who had experience in other care settings (eg, hospital pharmacies). The generalizability of our findings to other clinical areas may be limited as information infrastructures, work organization, culture, or environmental conditions and tasks vary across facilities and jurisdictions.

### Conclusions

A tremendous opportunity exists to leverage recent innovations in digital technologies to improve ADR reporting by CPs. To ensure successful uptake, we recommend that future reporting systems are provider focused and user-friendly. Furthermore, these systems should be integrated within the clinical workflow, enabling documentation and information sharing with regulators, allied health providers, and consumers. A comprehensive and multifaceted approach to systems design, implementation, and evaluation may improve adoption and ADR reporting. Importantly, these approaches must allow for meaningful engagement with clinician-users in the design, evaluation, and implementation phases and should include observational methods to identify differences between the actual and perceived use of ADR reporting systems. The framework outlined in this paper offers an example of how a socio-technical framework and a UCD approach may be integrated in an iterative fashion throughout the different stages of the intervention-design-cycle to meet this need, from analysis to deployment. In the future, it will be interesting to evaluate the success of such a framework and other theory-driven intervention strategies in terms of ADR reporting rates, patient safety, and health outcomes.

## Appendix

Multimedia Appendix 1 Human-centered design processes.

Multimedia Appendix 2 Structure of human activity in activity theory.

Multimedia Appendix 3 Proposed framework for leveraging activity theory within the user-centered design of ADR reporting systems.

## Abbreviations

ADR: adverse drug reaction
AT: activity theory
CP: community pharmacist
HP: health professional
PV: pharmacovigilance
TGA: Therapeutic Goods Administration
TTF: task-technology fit
UCD: user-centered design
