# Supplementary figures and images for: Linking Activity Theory Within User-Centered Design: Novel Framework to Inform Design and Evaluation of Adverse Drug Reaction Reporting Systems in Pharmacy
Source: JMIR Hum Factors. 2023 Feb 24;10:e43529. doi: 10.2196/43529 (PMC10007010; doi:10.2196/43529)

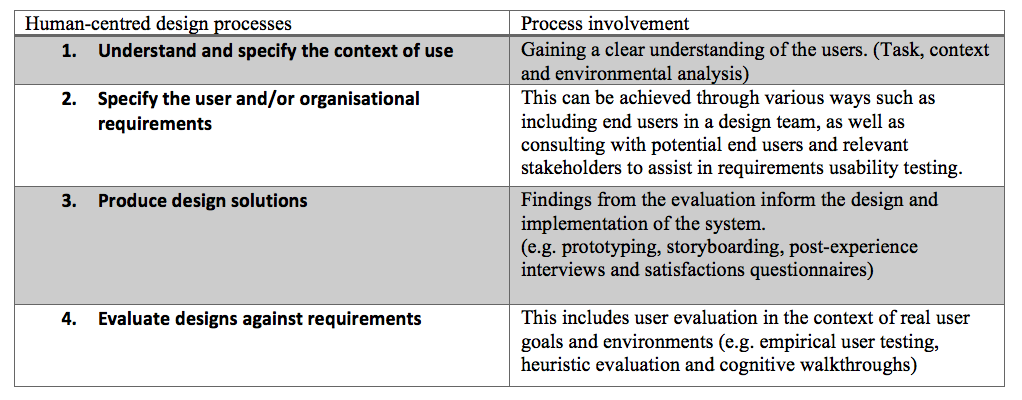

Supplement: Multimedia Appendix 1 [file humanfactors_v10i1e43529_app1.png]

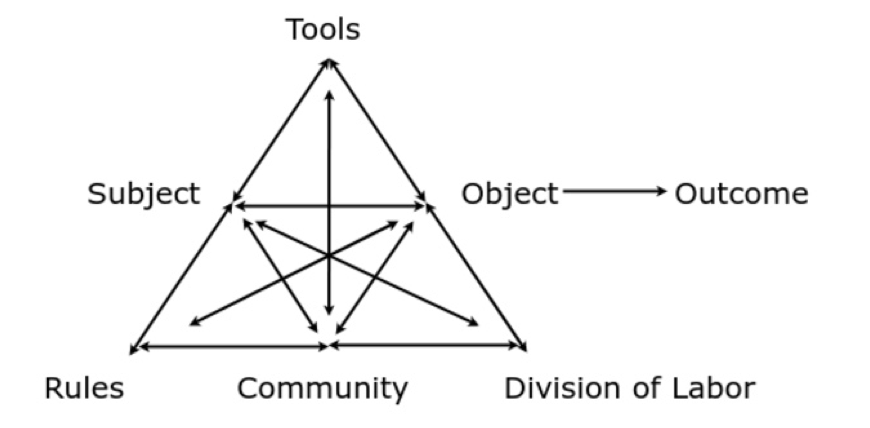

Supplement: Multimedia Appendix 2 [file humanfactors_v10i1e43529_app2.png]

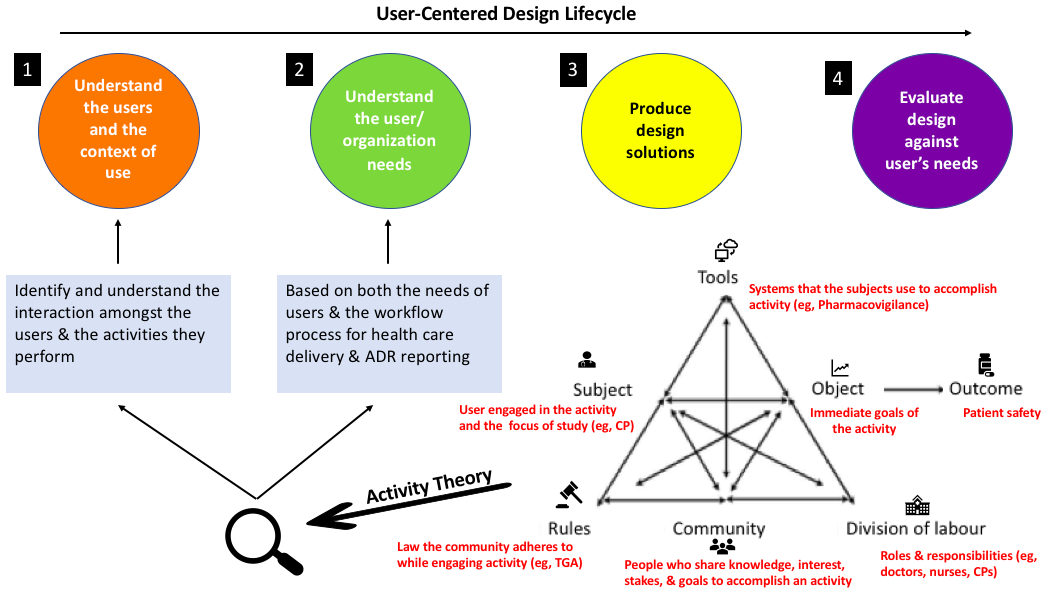

Supplement: Multimedia Appendix 3 [file humanfactors_v10i1e43529_app3.png]
